# Antibiotic Residues and Zinc Concentrations in the Livers and Kidneys of Portuguese Piglets—Relationship to Antibiotic and Zinc Resistance in Intestinal *Escherichia coli*

**DOI:** 10.1007/s12011-023-04032-0

**Published:** 2023-12-26

**Authors:** Olga Cardoso, Gabriela Assis, Maria M. Donato, Sara Carolina Henriques, Andreia Freitas, Fernando Ramos

**Affiliations:** 1 Faculdade de Farmácia, Universidade de Coimbra, CIEPQPF, Azinhaga de Santa Comba, 3000-548 Coimbra, Portugal; 2 Laboratório de Controlo da Alimentação Animal, Unidade Estratégica de Investigação E Serviços, Tecnologia E Segurança Alimentar, Instituto Nacional de Investigação Agrária E Veterinária, I.P, Av. da República, Quinta Do Marquês, 2780-157 Oeiras, Portugal; 3 Faculdade de Medicina, Universidade de Coimbra, CIMAGO, Azinhaga de Santa Comba, 3000-548 Coimbra, Portugal; 4 Faculty of Pharmacy, Universidade de Lisboa, Research Institute for Medicines (iMed.ULisboa), 1649-003 Lisbon, Portugal; 5 Laboratório Nacional de Referência Para a Segurança Alimentar, Instituto Nacional de Investigação Agrária E Veterinária, I.P., Rua Dos Lágidos, Lugar da Madalena, 4485-655 Vairão, Vila Do Conde, Portugal; 6 REQUIMTE/LAQV, Rua Dom Manuel II, Apartado 55142, 4051-401 Porto, Portugal; 7 Faculdade de Farmácia, Universidade de Coimbra, Azinhaga de Santa Comba, 3000-548 Coimbra, Portugal

**Keywords:** Zinc, Antibiotic resistance, *Escherichia coli*, Piglet farming, Zn tolerance, One Health

## Abstract

Metal ions such as zinc and copper have been used as alternatives to antibiotics, to improve animal health and growth rates in pig farming. This study aims to determine antibiotic residues and Zn concentration in piglets’ livers (*n* = 56) and kidneys (*n* = 60); and to examine the correlation between the use of Zn and antibiotics, and resistance to Zn and antibiotics of *Escherichia coli* isolated from piglets’ faeces (*n* = 60). Samples were collected from randomly selected healthy piglets (*n* = 60); antibiotic residues were quantified by ultra-high-performance-liquid-chromatography time-of-flight mass spectrometry (UHPLC-ToF–MS); Zn was quantified using flame atomic absorption spectrometry (FAAS); microbiological methods were used for *E. coli* isolation, antibiotic susceptibility, and Zn minimal inhibitory concentration; and Real-Time PCR was used for gene detection. The presence of antibiotic residues and Zn concentrations in the liver was found to be negatively correlated, whilst no significant difference was observed in the kidney. In *E. coli* isolated from piglet faeces considered to be susceptible or multi-drug-resistant, no significant difference was found between Zn concentrations in the liver and in the kidney, which appears to indicate that Zn accumulated in the liver and in the kidney does not promote resistance to antibiotics in *E. coli.* The isolates showed tolerance to Zn which would suggest that antibiotic resistance and phenotypic tolerance to Zn in these isolates are not related. The genes *zit*B and *znt*A associated to Zn tolerance, were predominantly found in the more resistant Zn isolates. The findings provide insights on how Zn use in pig production maintains antibiotic resistance and metal tolerance in bacteria, with implications for One Health.

**Supplementary Information:**

The online version contains supplementary material available at 10.1007/s12011-023-04032-0.

## Introduction

Antibiotics have been used worldwide to treat livestock diseases and as growth promoters, but the overuse of antibiotics has lead to the emergence of highly resistant bacteria [1–3]. In 2006, the European Commission banned the use of antibiotics as growth promoters [4]. Metal ions including zinc and copper, have been suggested as an alternative to improve animal health and growth rates [5, 6].

Zn is an essential trace element for all forms of life, performing several biological functions at low concentrations, but it is toxic at high concentrations. Zn has a ubiquitous cellular distribution and is an important structural component and a regulatory co-factor of a wide range of different enzymes in several important biochemical pathways in both plants and animals. Organisms have developed a homeostatic capacity that allows them to regulate the internal concentration of essential elements to a certain extent and to maintain it at optimal levels under varying external availabilities [7, 8]. Some authors have reported that after a period of high dietary Zn followed by dietary Zn reduction, the concentration of Zn in the tissues returns to normal values after two weeks, indicating that the accumulation of Zn is reversible after this period [9, 10]. The nutritional requirement for Zn in weaned piglets is 80—100 mg/kg but levels of 2500–3000 mg/kg were found in two–three week post-weaning dietary supplies used to reduce diarrhoea in piglets [6, 11, 12], one of the most common threats in the pig industry. Overuse and misuse of metals has led to their accumulation in the environment, which could in turn lead to antibiotic-resistant bacteria through co-selection [2, 6, 13]. This has led the European Union to legislate on the use of ZnO for veterinary applications in 2017 [14] and on its prohibition as growth promoter in 2022.

Indicator organisms of the animal gut microbiota can be used to assess the impact of antibiotics in pig production. The bacterium *Escherichia coli* is a widely used indicator organism due to its commensal nature and abundance in the gut. Acquired resistance to antibiotics is common in *E. coli* and as genes encoding antibiotic resistance traits can be transferred between commensal and pathogenic Enterobacteriales, the resistance pattern in *E. coli* is thought to represent most of the resistance traits found in Gram-negative bacteria in animals [3].

Organisms have had to develop strategies to deal with the toxic effects of Zn. Bacteria tightly control the uptake and efflux of Zn to ensure proper cellular function while avoiding metal toxicity. Free Zn in the cytoplasm is essentially absent, since the Zn regulators ZntR (efflux) and Zur (uptake) respond to free Zn concentrations of 10^−16^ M in *E. coli* [13, 15, 16].

Zn resistance in bacteria is facilitated via efflux [13, 15]. At least four systems involved in transporting Zn out of the cell have been identified [13]. These systems can be encoded chromosomally or/and in plasmids. The best characterised member of the efflux group in *E. coli* is ZntA, which is chromosomally encoded by the *znt*A gene. A second family of membrane transporters associated with bacterial Zn export are the cation diffusion facilitator proteins. *E. coli* contains two genes, *zit*B and *fie*F, which encode the proteins involved in Zn transport [13]. While deletion of *zit*B alone does not affect the ability of *E. coli* to handle high concentrations of Zn, a doubly-defective-mutant in *zit*B and *znt*A shows higher Zn sensitivity than a mutant strain defective in *znt*A alone [13]. It has been suggested that ZitB is involved in maintaining Zn homeostasis under 'normal' conditions, while ZntA confers Zn resistance [13, 15, 16].

Enteral bacteria in farm animals have been shown to develop resistance to trace elements. Some authors have concluded that antibiotics and metals used in animal husbandry can promote the spread of bacteria resistant to these stressors due to genetic and physiological links between the two resistances [7, 17, 18]. Zn appears to have the potential to exert a selective pressure that leads to increased Zn and antibiotic resistance in farms and in the environment [7].

Although the EU prohibited the veterinary use of ZnO in 2017, with effect from 2022 [14], no published studies have been found on possible relationships between Zn levels and antibiotic residues in piglet viscera; and in *E. coli* isolates susceptibility to antibiotics and tolerance to Zn. The authors of the present study consider research in this area to be invaluable particularly in the context of the ‘One Health’ approach.

Therefore, the aims of the present study are as follows: To determine the levels of antibiotic residues and Zn concentration in the liver and kidneys of Portuguese piglets; to ascertain whether there is a negative correlation between the use of Zn and the use of antibiotics as growth promoters; and to ascertain whether there is a correlation between the use of antibiotics and of Zn, and resistance to antibiotics and Zn in *E. coli* isolated from piglets’ faeces, as a representative of the intestinal microbiota.

## Materials and Methods

### Sampling and Isolation of *E. coli*

In this study samples of faeces (*n* = 60), liver (*n* = 56) and kidney (*n* = 60) were taken from randomly selected healthy piglets (*n* = 60; weighing 5—8 kg), between October 2018—May 2019. The piglets were obtained from a slaughterhouse in Mealhada that sources piglets from different regions of Portugal, due to the high demand for roast piglet ("leitão à bairrada"), a local gastronomic delicacy [19]. Sampling was performed under the supervision of a veterinarian..

The 60 piglets came from 8 different farms. Fourteen piglets from Aveiro were sampled (five in October; five in November; four in December); ten piglets from Pombal were sampled (five in March; five in April); nine piglets from Portela were sampled (five in March; four in May); nine piglets from Faro were sampled(five in March; four in May); 5 piglets from Alcobaça were sampled in January; five piglets from Batalha were sampled in February; four piglets from Caldas da Rainha were sampled in February; and four piglets from Castelo Branco were sampled in December. This study was carried out within the transition period of the EU banning ZnO for veterinary use [14].

Samples collected from each animal consisted of a minimum of 10 g of faeces; 200 g of liver (right lobe); and one whole kidney. Each sample was stored in a plastic bag and immediately transported to the laboratory. Liver and kidney samples were stored at -18 °C until analysis.

Processing for the isolation of *E. coli* was carried out on the day of collection using lauryl sulphate agar. Up to six morphologically different *E. coli* colonies were selected to ensure the collection of a variety of *E. coli* strains (Table EMS 1) [19].

### Zn quantification and Allocation of Levels in Liver and Kidney

The quantification of Zn in liver and kidney samples was obtained by FAAS using an air-acetylene flame, after dry-ashing, following an internal method based on ISO 6869:2000 [20] and ISO 14082:2003 [21]. The spectrometer used was a Thermo Scientific iCE 3000 with single-element hollow cathode lamps.

Each sample was processed separately as follows. Each sample was homogenized; 5 g (fresh weight) were placed in vitrosil crucibles and dry-ashed at 450 ºC in a muffle furnace, with gradual temperature increase; the dry-ash was dissolved in hydrochloric acid 0.6 M and solutions obtained to be read within the range of the calibration curve for Zn; the solutions were transferred to the nebulizer, where the element was sucked and atomized by flame action fed with acetylene and compressed air, emitting light at 213.9 nm for Zn.

The Zn content was obtained from a calibration curve. A total of 5 calibration standards were prepared by diluting a commercially available Zn standard solution (1000 mg/L, Merck) in hydrochloric acid. Class A 100 mL volumetric flasks were used, to which 25 µL, 50 µL, 75 µL, 100 µL and 125µL of the Zn commercial standard solution were transferred, and the remaining volume completed with 0.6 M hydrochloric acid solution. A zero point was performed with the reagent alone. Different standards of Zn were used to ensure analytical quality control. Quality control of the calibration curves was performed by reading two further standard solutions, with concentrations near the lowest and the highest values of the curve, prepared from ZnCl (Supelco). The preparation was based on the molecular weight of the substance containing the element, and a stock solution of 1000 mg/L was obtained. Two solutions that read near the extremes of the calibration curve were obtained. The first control was read at the beginning of the day, and the second control was read at the end of the day (tolerance of 90 -110%).

The linearity of the curve was assessed by means of its graphical representation together with the analysis of the correlation coefficient, which was greater than 0.999 in all the curves used for the values obtained.

The samples were analyzed in duplicate and the final result was the average of the two measurements obtained.

Values of Zn in the liver were classified according to López-Alonso [22]: deficient; marginal (15—30 mg/kg); adequate (35—90 mg/kg); high (above 200 mg/kg); and toxic (500—3100 mg/kg). Due to the gap 90—200 mg/kg and the lack of differentiation down to the lower toxic concentration in these definitions, in the present study, the "high level" is redefined to include values from 90 to 500 mg/kg.

Regarding levels of Zn concentration in the kidneys two levels were classified [22]: adequate (15—30 mg/kg) and toxic (190—367 mg/kg). In the present study a new level, the “high-level” (30—190 mg/kg) is proposed.

### Quantification of Antibiotic Residues

Forty-three antibiotics from seven therapeutic classes were determined by UHPLC-ToF–MS (Table EMS 2). Antibiotic standards and internal standards with purity ≥ 98%, were purchased from Sigma-Aldrich (Madrid, Spain).The extraction procedure and the UHPLC-ToF–MS conditions were previously described [23].

### Antibiotic and Zn Susceptibility Assays

Minimum inhibitory concentrations (MIC) for Zn, defined as the lowest concentration of Zn at which no visible growth was observed, were determined by the agar dilution method, applying standard bacteriological methods. ZnCl_2_ stock solution was added to molten Mueller–Hinton (MH) agar to obtain final ZnCl_2_ concentrations of 0.5–32 mM [5, 15, 16, 24–26]. A culture of each isolate was diluted to 1 × 10^7^ CFU/mL of which.

1 µL was inoculated as spots with a microplate replicator, followed by overnight incubation at 37 °C; each assay was performed in triplicate; and the plates without Zn were used as controls.

Isolates were tested for antibiotic susceptibility by agar disk diffusion on MH agar following both the European Committee on Antimicrobial Susceptibility Testing guidelines [27] and Cardoso et al. [19]. The following antibiotics (Liofilchem®s.r.l., Italy) were used. Seven classes of beta-lactams: imipenem (IP) (10 µg) (carbapenem); aztreonam (AZT) (30 µg) (monobactam); piperacillin (PIP) (30 µg) (ureidopenicillin); amoxicillin-clavulanic acid (AMC) (20–10 µg) (penicillin + beta-lactamase inhibitor); cefoxitin (FOX) (30 µg) (2nd generation cephalosporins); ceftazidime (CAZ) (10 µg) (3th generation cephalosporin); and cefepime (FEP) (30 µg) (4th generation cephalosporin). The remaining 3 antibiotics were from other families including amikacin (AK) (30 µg) (aminoglycoside); ciprofloxacin (CIP) (5 µg) (fluoroquinolone); and sulfamethoxazole-trimethoprim (SXT) (23.75 – 1.25 µg) (anti-metabolite).

The definition of multidrug-resistance (MDR) adopted in this study is the one of the European Centre for Prevention and Disease Control (ECDC) published by Magiorakos [28]. MDR “acquired non-susceptibility to at least one agent in three or more antimicrobial categories”; extensively drug-resistance (XDR) “non-susceptibility to at least one agent in all but two or fewer antimicrobial categories”; and pandrug-resistance (PDR) “non-susceptibility to all agents in all antimicrobial categories”.

### Determination of Zn Tolerance Genes

Zn tolerance genes were determined by Real-Time PCR (LightCycler, Roche Diagnostics, Germany) on crude bacterial DNA (Table 1).

**Table 1:** Specific primers and PCR conditions used in amplification of Zn tolerance genes

Gene	Primer sequence (5’–3’)	PCR conditions	Product length (bp)	Reference
*zit*B	TAC GAC GCT TCA GTT CAG C CAC TTT CGG TTG GCT AAG AC	95 °C,10 s;54 °C,5 s;72 °C,18 s	449	[16]
*znt*A	CGA CGG TAA ACT GCT CTC AC CAA CGC AAT GAC ACC AAG	95 °C,10 s;53 °C,5 s;72 °C,34 s	859	[16]

PCR was performed in a volume of 20 µl containing 4.0 µl of LightCycler FastStart DNA MasterPLUS SYBR Green I® (Roche Diagnostics, Mannheim, Germany). An initial denaturation cycle at 95 °C for 10 min was performed in all cases followed by 45 amplification cycles. Table 1 shows the denaturation, annealing and extension conditions for each set of primers. Melting curves were plotted automatically and analysed (LightCycler software). PCR products were checked on 2% agarose gels stained with ethidium bromide and visualized with UV light. By comparing both results, it was possible to establish specific melting temperatures to identify each gene; positive and negative controls were included. To avoid errors, the procedures were separately repeated by different technicians.

### Statistical Analysis

Liver and kidney samples were categorized by the absence or presence of antibiotic residues.

Quantified Zn concentrations were summarized by mean and 95% confidence interval (CI) of the mean.

Welch’s two-sample *t*-test was performed to compare the Zn concentration in liver and kidney with absence and presence of antibiotic residues. Likewise, Welch’s two-sample *t*-test was used to detect differences in Zn concentration in liver and kidney in function of the *E. coli* identified in faeces considered as S or MDR.

The chi-squared test was used to test for differences in proportion of Zn MIC in function of the *E. coli* considered as S or MDR.

Further, the binomial test was used to compare the observed frequencies of the presence and absence of genes associated with bacterial tolerance to Zn in *E. coli* considered as MDR.

All results were evaluated at a 5% significance level.

Statistical and graphical analyses were performed in R version 4.2.1.

## Results and Discussion

### Quantification of Zn and Antibiotics Residues

Zn quantification and antibiotic residues (Table ESM 2) were determined in the livers of 56 piglets and in the kidneys of 60 piglets. A formal estimation of sample size was not performed. Nevertheless, following a post hoc analysis, 56 samples were sufficient to detect differences of at least 45 mg/kg, with a power of at least 80%, considering the observed standard deviation of approximately 300 mg/kg.

### In the Liver

A toxic level of Zn was detected in 13 livers (23.2%); the high level in 26 livers (46.4%), and an adequate level in 17 livers (30.4%). No deficient or marginal levels were detected. Regarding antibiotic residues in the liver, 32 livers showed no residues; 18 livers showed one or two residues; and six livers showed three or more residues.

One of the aims of the present study is to correlate the presence or absence of antibiotic residues to Zn levels.

In the livers of the 32 piglets without antibiotic residues, the mean Zn concentration was 440.6 mg/kg (95% CI: [312.9—568.3] mg/kg); a toxic level of Zn was observed in 13 livers; a high level of Zn was observed in 11 livers; and an adequate level of Zn was observed in 8 livers. In the remaining 24 livers where antibiotic residues were present, the mean Zn concentration was 156.6 mg/kg (95% CI: [113—200.2] mg/kg); toxic levels of Zn were not observed; a high level of Zn was observed in 15 livers; and an adequate level of Zn was observed in nine livers.

**Fig. 1:**
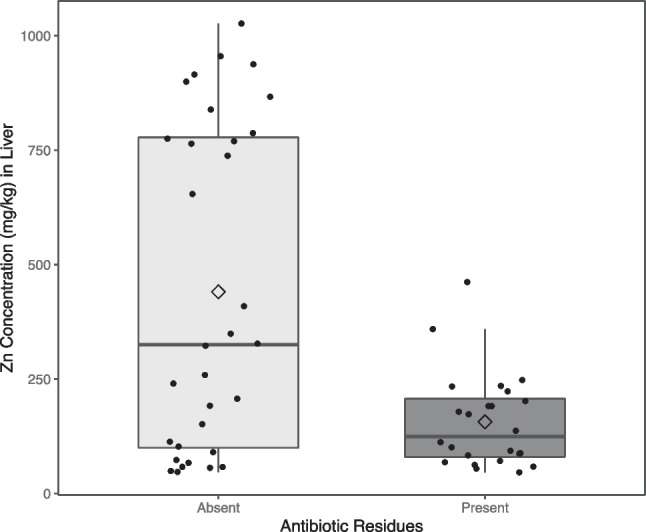
Box plot of Zn concentration (mg/kg) in liver against the absence or presence of antibiotic residues in piglets’ livers

Figure 1 shows a box plot of the distribution of Zn concentration by the absence or the presence of antibiotic residues in liver where dots represent individual values, and diamond shapes represent mean values for each group.

In piglets’ livers, the mean Zn concentration in livers with no antibiotics present is 284 mg/kg (95% CI: [150.3 – 417.8] mg/kg) higher than in livers with presence of antibiotic residues (*p*-value < 0.001), showing that there is a negative correlation between the Zn concentration and the presence of antibiotic residues.

### In the Kidney

In the 60 kidneys evaluated, five kidneys (8.3%) presented a toxic level; 16 kidneys (26.7%) presented a high level; and 39 kidneys (65%) presented an adequate level.

Regarding antibiotic residues, 31 kidneys presented no residues, with a mean Zn concentration of 44.3 mg/kg (95% CI: [25.4—63.3] mg/kg); one kidney presented a toxic level; eight kidneys presented a high level; and 22 kidneys presented an adequate level. The remaining 29 kidneys presented at least one antibiotic residue, with a mean Zn concentration of 69.9 mg/kg (95% CI: [41.4—98.4] mg/kg); four kidneys presented the toxic level; eight kidneys presented the high level; and 17 kidneys presented the adequate level.

**Fig. 2:**
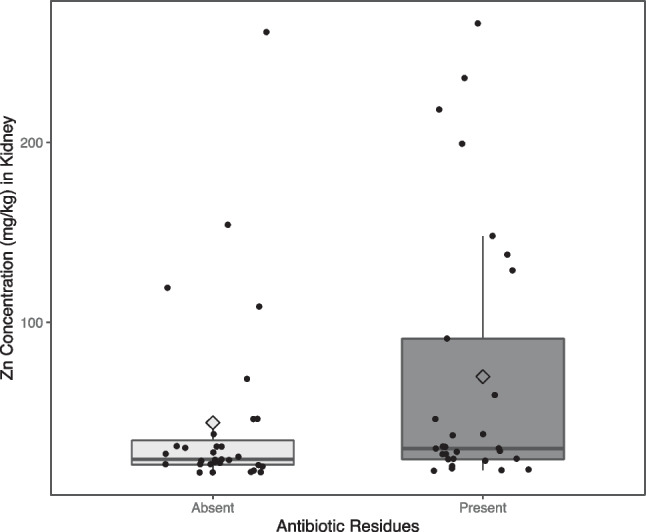
Box plot of Zn concentration (mg/kg) against the absence or presence of antibiotic residues detected in kidney

Figure 2 shows a box plot of the distribution of Zn concentration (mg/kg) in kidney by the absence or presence of antibiotic residues found in kidney, where dots represent individual values, and diamond shapes represent mean values for each group.

No significant differences were found in Zn concentrations between kidneys with absence of antibiotic residues when compared to kidneys with a presence of antibiotic residues (-25.6 mg/kg [-59.2 – 8] mg/kg, *p*-value = 0.133).

The Zn concentrations found in a study of intensive farming in Galicia [22] were in the adequate level which is in contrast with the findings of the present study.

The Zn concentration in liver and kidney and the presence or absence of antibiotic residues, show a negative correlation in the liver (*p*-value < 0.001) and no significant difference in the kidneys (*p*-value = 0.133). The results suggest that high dosages of Zn may have been administered before the sampling, which could have led to a build-up of Zn in piglets’ livers. In farms where multiple collections were carried out at different times of year, both the Zn levels and the antibiotic residues were consistent.. For example, in Faro where the two samples taken two months apart in March and May the first sample showed high levels of Zn in both the liver and kidney; no antibiotic residues in the liver; and only one residue in the kidney. No published research on Zn build-up and antibiotic residues in piglets’ kidneys and livers was found that could be compared to the present study.

The results on Zn in the present study could indicate an increased use of metal ions in recent years. Such increased use could lead to further environmental contamination, and to the emergence of select bacteria exhibiting higher resistance to both antibiotics and to metal ions [13, 17, 18, 29, 31]. Since the removal of Zn from veterinary use in 2017 [14], alternatives began to be considered including probiotics, prebiotics, organic acids and essential oils [32].

### Correlation of Antibiotic Susceptibility of *E. coli* Isolates and Zn Concentration

**Fig. 3:**
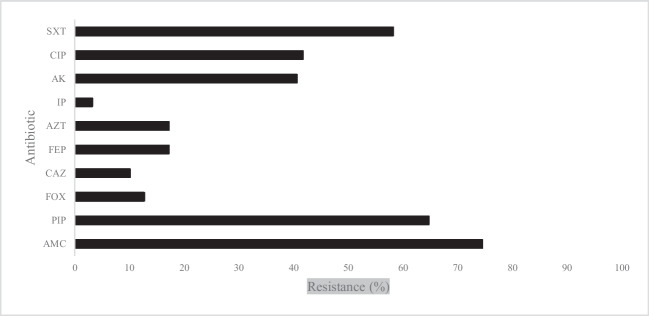
Antibiotic resistance (%) of *E. coli* isolates to imipenem (IP); aztreonam (AZT); piperacillin (PIP); amoxicillin-clavulanic acid (AMC); cefoxitin (FOX); ceftazidime (CAZ); cefepime (FEP); amikacin (AK); ciprofloxacin (CIP); and sulfamethoxazole-trimethoprim (SXT)

The susceptibility to antibiotics used in human health was determined to observe the resistance of 276 *E. coli* isolates. The results are shown in Fig. 3. The highest resistance was determined for amoxicillin and acid clavulanic (74.5%), followed by piperacillin (64.7%); trimethoprim and sulphamethoxazole (58.2%); ciprofloxacin (41.7%); amikacin (40.6%); The remaining beta-lactams used in this study showed resistance levels below 20%, whereas 3.2% were resistant to imipenem. *E. coli* isolates were more resistant to these agents than those reported in previous studies on EU countries including Portugal [33–35].

**Fig. 4:**
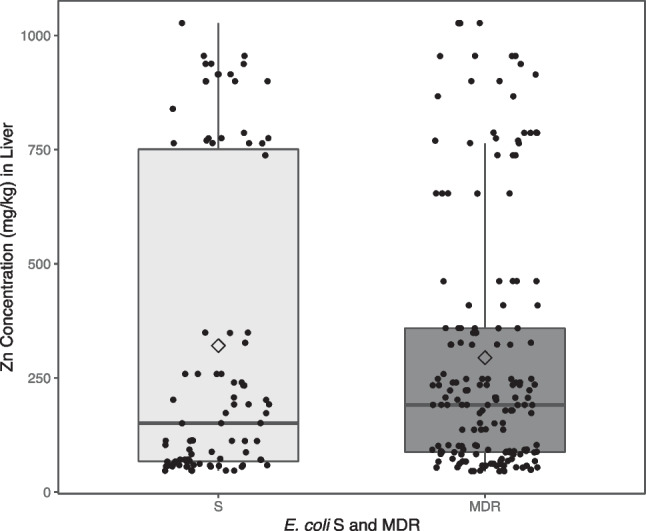
Box Plot of Zn concentration against *E. coli* considered as S or MDR

The 276 *E. coli* isolates were distributed in three resistance profiles, of which 35.2% were susceptible (S) to 8 or more antibiotics; 61.2% were MDR; 3.2% were XDR; and 0.4%were PDR. The high resistance found in farmed animals has been previously reported in countries worldwide [34, 36, 37].

### In the Liver

The antibiotic susceptibility correlation to Zn concentration in liver was tested. MDR was observed in 169 (65%) *E. coli*, and the mean of Zn concentration was 294.4 mg/kg (95% CI: [252.4 – 336.5] mg/kg); Ninety-one (35%) were considered susceptible (S) to most of the antibiotics tested and the mean of Zn concentration was 320.7 mg/kg (95% CI: [250.9 – 390.4] mg/kg).

Figure 4 shows a box plot distribution of Zn concentration in liver, by the *E. coli* considered as S or MDR, where dots represent individual values, and diamond shapes represent mean values for each group.

No significant differences were found in Zn concentration between *E. coli* considered as S or MDR (26.2 mg/kg [-54.9 – 107.3] mg/kg, *p*-value = 0.524).

### In the Kidney

A total of 276 *E. coli* samples was isolated from the faeces of 60 piglets, where 96 were considered as S, and the mean Zn concentration in kidney was 60.0 mg/kg (95% CI: [45.8 – 74.2] mg/kg). One hundred eighty *E. coli* isolates were considered MDR, with the mean of Zn concentration in kidney of 48.8 mg/kg (95% CI: [40.9 – 56.8] mg/kg).

**Fig. 5:**
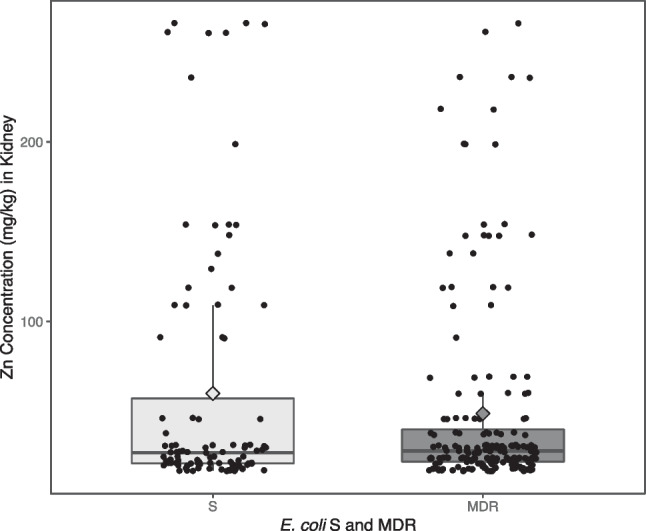
Box Plot of Zn Concentration (mg/kg) by the *E. coli* considered as S or MDR

The Fig. 5 is a box plot of the distribution of Zn concentration (mg/kg) in kidney, by the *E. coli* considered as S or MDR. Individual values are presented in dots, and the mean value for each group is represented in diamond shape.

No significant differences were found between Zn concentration and *E. coli* considered as S or MDR (11.2 mg/kg [-5 – 27.4] mg/kg, *p*-value = 0.176).

In bacteria isolated from piglet faeces and considered S or MDR, there was no statistical difference with Zn concentrations in either liver or kidney, but a positive correlation between Zn concentration and antibiotic resistance has been observed in other matrices such as manure [31] and in piglets supplemented with Zn [30].

### Correlation of Zn MIC and Antibiotic Susceptibility in *E. coli* Isolates

Regarding Zn MIC, 257 (93%) isolates had a MIC of 8 mM and 19 (7%) had a MIC of 4 mM. 66% of *E. coli* with a MIC of 8 mM, were MDR and 34% were S; 42% of *E. coli* with a MIC of 4 mM, were S and 58% were MDR. A trend towards a higher probability of MDR *E. coli* with a Zn MIC of 8 mM was observed. Due to the small sample size of *E. coli* with a Zn MIC of 4 mM, no statistical differences were found between *E. coli* isolates categorised as S and MDR as a function of Zn MIC (p-value = 0.4874).

Irrespective of antibiotic susceptibility or resistance, the isolates showed tolerance to Zn, as the majority had a Zn MIC of 8 mM, suggesting that antibiotic resistance and phenotypic tolerance to Zn in these isolates may be unrelated [5]. In this study, it was not possible to determine whether the piglets had been previously supplemented with Zn. However, the high and toxic levels observed in both liver and kidney indicated that Zn might have been previously used as a growth promoter. Excess Zn is excreted to the gut, stressing the intestinal microbiota, and is eliminated through the faeces, contaminating manure and the environment [29, 31]. This hypothesis is reinforced by the fact that the *E. coli* isolates in this study showed a much higher tolerance to Zn (8 mM) than the values previously reported by other authors [5, 15, 16, 24, 30, 31].

### Zn Tolerance in *E. coli* Isolates

The *zit*B and *znt*A genes, which are associated with bacterial tolerance to Zn, were detected by real-time PCR. The *zit*B gene was observed in 230 (83%) of the isolates and the *znt*A gene in 140 (51%) of the isolates. *E. coli* with *zit*B gene were more likely to have a Zn MIC of 8 mM [214 (93%)] than a Zn MIC of 4 mM [16 (7%)] (*p*-value < 0.0001). When the *znt*A gene was present, bacteria were also more likely to have a Zn MIC of 8 mM [129 (92.1%)] than a Zn MIC of 4 mM [11 (7.9%)], (*p*-value < 0.0001).

The association of the two genes was present in 116 (42%) of the isolates. These *E. coli* had a higher probability of having a Zn MIC of 8 mM [106 (91.4%)] than of having a Zn MIC of 4 mM [10 (8.6%)], (*p*-value < 0.0001).

To ensure proper cellular function while avoiding metal toxicity, bacteria regulate the uptake and efflux of Zn. The presence of genes related to Zn detoxification and bacterial tolerance to Zn, such as *zit*B and *znt*A, is ubiquitous [13, 15]. The presence of these Zn-tolerance-related genes, mainly in the more resistant Zn isolates (MIC of 8 mM), was also demonstrated in this study, in agreement with previous work [15, 16, 38].

## Conclusions

This work showed that when there was a high concentration of Zn in the piglets’ liver there was no detection of antibiotic residues, and vice versa.

The antibiotic susceptibility profiles of the *E. coli* isolates were not related to the Zn concentrations in either the piglets' liver or kidneys.

*E. coli* isolates were mostly resistant to antibiotics and tolerant to Zn (MIC 8 mM, and the presence of the ubiquitous *znt*A and *zit*B), and these two variables were not correlated. It could be that these bacteria had been already exposed to antibiotic pressure previous to the present study, which could have triggered multidrug resistance. In the presence of Zn they could have adapted more easily to this stressor and they could have become more resistant to it, with implications for ‘One Health’.

### Supplementary Information

Below is the link to the electronic supplementary material.

Supplementary file1 (PDF 358 KB)

Supplementary file2 (PDF 264 KB)

Supplementary file3 (PDF 197 KB)

## Data Availability

All the data and tools/models used for this work are publicly available.
