# Supplementary material for: Antibiotic Residues and Zinc Concentrations in the Livers and Kidneys of Portuguese Piglets—Relationship to Antibiotic and Zinc Resistance in Intestinal Escherichia coli
Source: Biol Trace Elem Res. 2023 Dec 26;202(10):4522–30. doi: 10.1007/s12011-023-04032-0 (PMC11339090; doi:10.1007/s12011-023-04032-0)
Supplement: Supplementary file 1 — Supplementary file1 (PDF 358 KB) [file 12011_2023_4032_MOESM1_ESM.pdf]

~~Antibiotics and Zinc in Piglet Farming: A Study on Antibiotic and Zinc Resistance in *Escherichia coli*~~

Antibiotic Residues and Zinc Concentrations in the Livers and Kidneys of Portuguese Piglets - Relationship to Antibiotic and Zinc Resistance in intestinal *Escherichia coli*

Biological Trace Elemental Research

Olga Cardoso [1], Gabriela Assis [2], Maria M. Donato [3*], Sara Carolina Henriques[4], Andreia Freitas [2,5], Fernando Ramos [6,7]

[1] Universidade de Coimbra, CIEPQPF, Faculdade de Farmácia, Azinhaga de Santa Comba, 3000-548 Coimbra, Portugal; ocardoso@ci.uc.pt; ORCID 0000-0002-8902-0213

[2] Laboratório de Controlo da Alimentação Animal, Unidade Estratégica de Investigação e Serviços, Tecnologia e Segurança Alimentar, Instituto Nacional de Investigação Agrária e Veterinária, I.P, Av. da República, Quinta do Marquês, 2780-157 Oeiras Portugal; gabriela.assis@iniav.pt

[3] Universidade de Coimbra, CIMAGO, Faculdade de Medicina, Azinhaga de Santa Comba, 3000-548 Coimbra, Portugal; mmdonato@fmed.uc.pt; ORCID 0000-0003-0543-0088

[4]Universidade de Lisboa, Research Institute for Medicines (iMed.ULisboa), Faculty of Pharmacy, 1649-003 Lisboa, Portugal; sarachenriques@ff.ulisboa.pt; ORCID: 0000-0001-9649-4823

[5] Laboratório Nacional de Referência para a Segurança Alimentar, Instituto Nacional de Investigação Agrária e Veterinária, I.P., Rua dos Lágidos, Lugar da Madalena, 4485-655 Vairão, Vila do Conde, Portugal; andreia.freitas@iniav.pt; ORCID 0000-0003-3292-5473

[6] REQUIMTE/LAQV, Rua Dom Manuel II, Apartado 55142, 4051-401 Porto, Portugal

[7] Universidade de Coimbra, Faculdade de Farmácia, Azinhaga de Santa Comba, 3000-548 Coimbra, Portugal; framos@ff.uc.pt; ORCID 0000-0002-6043-819X

* Correspondence: mmdonato@fmed.uc.pt; ORCID 0000-0003-0543-0088

Table ESM1 Number of *E. coli* isolated from each piglet

Piglet Identification	*E. coli* (N=)
16	4
17	3
18	3
19	4
20	4
21	4
22	4
23	4
24	4
25	4
26	4
27	4
28	4
29	4
30	4
31	4
32	4
33	4
34	4
35	3
36	6
37	5
38	6
39	4
40	6
41	5
42	4
43	6
44	4
45	6
46	6
47	6
48	6
49	5
50	5
51	5
52	5
53	5

54	5
55	3
56	5
57	4
58	6
59	6
60	6
61	1
62	6
63	6
64	4
65	4
66	6
67	6
68	2
69	1
70	4
71	6
72	6
73	5
74	6
75	6
