# Supplementary material for: Antibiotic Residues and Zinc Concentrations in the Livers and Kidneys of Portuguese Piglets—Relationship to Antibiotic and Zinc Resistance in Intestinal Escherichia coli
Source: Biol Trace Elem Res. 2023 Dec 26;202(10):4522–30. doi: 10.1007/s12011-023-04032-0 (PMC11339090; doi:10.1007/s12011-023-04032-0)
Supplement: Supplementary file 2 — Supplementary file2 (PDF 264 KB) [file 12011_2023_4032_MOESM2_ESM.pdf]

~~Antibiotics and Zinc in Piglet Farming: A Study on Antibiotic and Zinc Resistance in *Escherichia coli*~~

Antibiotic Residues and Zinc Concentrations in the Livers and Kidneys of Portuguese Piglets - Relationship to Antibiotic and Zinc Resistance in intestinal *Escherichia coli*

Biological Trace Elemental Research

Olga Cardoso [1], Gabriela Assis [2], Maria M. Donato [3*], Sara Carolina Henriques[4], Andreia Freitas [2,5], Fernando Ramos [6,7]

[1] Universidade de Coimbra, CIEPQPF, Faculdade de Farmácia, Azinhaga de Santa Comba, 3000-548 Coimbra, Portugal; ocardoso@ci.uc.pt; ORCID 0000-0002-8902-0213

[2] Laboratório de Controlo da Alimentação Animal, Unidade Estratégica de Investigação e Serviços, Tecnologia e Segurança Alimentar, Instituto Nacional de Investigação Agrária e Veterinária, I.P, Av. da República, Quinta do Marquês, 2780-157 Oeiras Portugal; gabriela.assis@iniav.pt

[3] Universidade de Coimbra, CIMAGO, Faculdade de Medicina, Azinhaga de Santa Comba, 3000-548 Coimbra, Portugal; mmdonato@fmed.uc.pt; ORCID 0000-0003-0543-0088

[4] Universidade de Lisboa, Research Institute for Medicines (iMed.ULisboa), Faculty of Pharmacy, 1649-003 Lisboa, Portugal; sarachenriques@ff.ulisboa.pt; ORCID: 0000-0001-9649-4823

[5] Laboratório Nacional de Referência para a Segurança Alimentar, Instituto Nacional de Investigação Agrária e Veterinária, I.P., Rua dos Lágidos, Lugar da Madalena, 4485-655 Vairão, Vila do Conde, Portugal; andreia.freitas@iniav.pt; ORCID 0000-0003-3292-5473

[6] REQUIMTE/LAQV, Rua Dom Manuel II, Apartado 55142, 4051-401 Porto, Portugal

[7] Universidade de Coimbra, Faculdade de Farmácia, Azinhaga de Santa Comba, 3000-548 Coimbra, Portugal; framos@ff.uc.pt; ORCID 0000-0002-6043-819X

* Correspondence: mmdonato@fmed.uc.pt; ORCID 0000-0003-0543-0088

Table ESM~~1~~2 Antibiotics and respective classes

Antibiotic	Antibiotic Class
Ampicillin	
Benzylpenicillin	Penicillin
Nafcillin	
Oxacillin	
Cefalonium	
Cefazolin	Cephalosporin
Cefquinome	
Ceftiofur	
Chlortetracyclin	
Dicloxacillin	
**Doxycycline**	
epi-Chortetracyclin	Tetracycline
epi-Tetracyclin	
**Oxytetracycline**	
Tetracycline	
Cinoxacin	
Nalidixic acid	
Oxolinic acid	
**Ciprofloxacin**	
**Danofloxacin**	
Enoxacin	Quinolone
**Enrofloxacin**	
Flumequine	
Marbofloxacin	
Norfloxacin	
Ofloxacin	
**Spiramycin**	
**Tilmicosin**	Macrolide
Tylosin A	
**Sulfachloropyridazine**	
**Sulfadiazine**	
Sulfadimethoxine	
Sulfadimidin	
Sulfadoxine	
Sulfamethizol	
Sulfamethoxazole	Sulfonamide
Sulfanilamide	
Sulfapyridin	
Sulfaquinoxaline	
Sulfathiazole	
**Sulfisomidine**	
Sulfisoxazole	
**Trimethoprim**	Trimethoprim

In bold the antibiotic residues detected
