# Supplementary material for: Antibiotic Residues and Zinc Concentrations in the Livers and Kidneys of Portuguese Piglets—Relationship to Antibiotic and Zinc Resistance in Intestinal Escherichia coli
Source: Biol Trace Elem Res. 2023 Dec 26;202(10):4522–30. doi: 10.1007/s12011-023-04032-0 (PMC11339090; doi:10.1007/s12011-023-04032-0)
Supplement: Supplementary file 3 — Supplementary file3 (PDF 197 KB) [file 12011_2023_4032_MOESM3_ESM.pdf]

~~Antibiotics and Zinc in Piglet Farming: A Study on Antibiotic and Zinc Resistance in *Escherichia coli*~~

# Antibiotic Residues and Zinc Concentrations in the Livers and Kidneys of Portuguese Piglets - Relationship to Antibiotic and Zinc Resistance in intestinal *Escherichia coli*

Biological Trace Elemental Research

Olga Cardoso [1], Gabriela Assis [2], Maria M. Donato [3*], Sara Carolina Henriques[4], Andreia Freitas [2,5], Fernando Ramos [6,7]

[1] Universidade de Coimbra, CIEPQPF, Faculdade de Farmácia, Azinhaga de Santa Comba, 3000-548 Coimbra, Portugal; ocardoso@ci.uc.pt; ORCID 0000-0002-8902-0213

[2] Laboratório de Controlo da Alimentação Animal, Unidade Estratégica de Investigação e Serviços, Tecnologia e Segurança Alimentar, Instituto Nacional de Investigação Agrária e Veterinária, I.P, Av. da República, Quinta do Marquês, 2780-157 Oeiras Portugal; gabriela.assis@iniav.pt

[3] Universidade de Coimbra, CIMAGO, Faculdade de Medicina, Azinhaga de Santa Comba, 3000-548 Coimbra, Portugal; mmdonato@fmed.uc.pt; ORCID 0000-0003-0543-0088

[4]Universidade de Lisboa, Research Institute for Medicines (iMed.ULisboa), Faculty of Pharmacy, 1649-003 Lisboa, Portugal; sarachenriques@ff.ulisboa.pt; ORCID: 0000-0001-9649-4823

[5] Laboratório Nacional de Referência para a Segurança Alimentar, Instituto Nacional de Investigação Agrária e Veterinária, I.P., Rua dos Lágidos, Lugar da Madalena, 4485-655 Vairão, Vila do Conde, Portugal; andreia.freitas@iniav.pt; ORCID 0000-0003-3292-5473

[6] REQUIMTE/LAQV, Rua Dom Manuel II, Apartado 55142, 4051-401 Porto, Portugal

[7] Universidade de Coimbra, Faculdade de Farmácia, Azinhaga de Santa Comba, 3000-548 Coimbra, Portugal; framos@ff.uc.pt; ORCID 0000-0002-6043-819X

* Correspondence: mmdonato@fmed.uc.pt; ORCID 0000-0003-0543-0088

Table ESM23 Zn concentration and antibiotics residues detected in kidney and liver piglets

| Piglet Number | Zn (mg/Kg) | | Antibiotic Residue | |
|---|---|---|---|---|
| | Kidney | Liver | Kidney | Liver |
| 16 | 26.84 | 190.78 | Tilmicosin | Doxycycline |
| 17 | 37.74 | 327.33 | Tilmicosin | |
| 18 | 26.78 | 191.52 | | |
| 19 | 29.92 | 89.91 | Tilmicosin;Sulfisomidine | |
| 20 | 28.58 | 102.84 | Tilmicosin | |
| 21 | 27.34 | 233.65 | Doxycycline | Doxycycline |
| 22 | 24.98 | 111.86 | | Enrofloxacin |
| 23 | 24.23 | 101.06 | | Doxycycline |
| 24 | 20.72 | 73.13 | | |
| 25 | 20.52 | 58 | | |
| 26 | 30.73 | 173.42 | Doxycycline;Sulfachloropyridazine;Sulfadiazine; Tilmicosin | Doxycycline; Tilmicosin;Trimethoprim |
| 27 | 36.52 | 222.94 | Doxycycline;Sulfachloropyridazine;Sulfadiazine; Tilmicosin | Doxycycline; Sulfachloropyridazine;Sulfadiazine; Tilmicosin |
| 28 | 23.53 | 67.66 | Doxycycline; Tilmicosin | Doxycycline; Tilmicosin;Trimethoprim |
| 29 | 28.37 | 201.81 | Doxycycline;Sulfachloropyridazine;Sulfadiazine; Tilmicosin | Doxycycline;Trimethoprim |
| 30 | 45.62 | 113.36 | | |
| 31 | 260.66 | 915.48 | | |
| 32 | 266.46 | 938.22 | Trimethoprim | |
| 33 | 90.81 | 774.84 | Trimethoprim | |
| 34 | 199.22 | 737.97 | Trimethoprim | |
| 35 | 137.79 | 770.39 | Trimethoprim | |
| 36 | 27.97 | 207.06 | | |
| 37 | 30.77 | 259.07 | | |
| 38 | 22.16 | 151.4 | | |
| 39 | 45.66 | 408.7 | | |
| 40 | 31.14 | 239.7 | | |

| | | | | |
|---|---|---|---|---|
| 41 | 23.54 | 248.23 | | Enrofloxacin |
| 42 | 20.15 | 46.43 | | Enrofloxacin; Ciprofloxacin |
| 43 | 16.8 | 93.04 | | Enrofloxacin; Ciprofloxacin |
| 44 | 24.27 | 178.63 | Enrofloxacin | Enrofloxacin; Ciprofloxacin |
| 45 | 30.01 | 348.92 | | |
| 46 | 17.06 | 70.61 | | Enrofloxacin; Ciprofloxacin; Danofloxacin; Spiramicyn |
| 47 | 17.61 | 83.07 | Enrofloxacin; Ciprofloxacin | Enrofloxacin; Ciprofloxacin |
| 48 | 17.19 | 86.57 | | Enrofloxacin |
| 49 | 21.66 | 235.07 | | Enrofloxacin; Ciprofloxacin; Danofloxacin; Spiramicyn |
| 50 | 19.14 | 87.62 | Enrofloxacin | Enrofloxacin |
| 51 | 59.77 | 461.91 | Enrofloxacin | Enrofloxacin; Ciprofloxacin |
| 52 | 29.58 | Nd | Enrofloxacin | Enrofloxacin; Ciprofloxacin |
| 53 | 30.67 | 190.55 | Enrofloxacin | Enrofloxacin |
| 54 | 24.11 | 137.49 | Enrofloxacin | Enrofloxacin; Ciprofloxacin |
| 55 | 17.44 | 49.06 | | |
| 56 | 37.63 | 322.94 | | |
| 57 | 23.29 | 57.71 | Oxytetracyclin | |
| 58 | 20.9 | 47.13 | | |
| 59 | 20.1 | 56.07 | Oxytetracyclin | |
| 60 | 17.68 | nd | Enrofloxacin | Enrofloxacin |
| 61 | 30.63 | nd | | |
| 62 | 17.63 | 62.38 | Enrofloxacin | Enrofloxacin; Ciprofloxacin; Danofloxacin |
| 63 | 45.52 | 359.32 | Enrofloxacin; Danofloxacin | Enrofloxacin |
| 64 | 23.02 | 59.03 | | Danofloxacin |
| 65 | 21.08 | nd | | |
| 66 | 17.92 | 67.06 | | |
| 67 | 23.54 | 53.69 | | Danofloxacin |
| 68 | 217.46 | 866.83 | Sulfadiazine; Trimethoprim | |
| 69 | 129.31 | 839.02 | Sulfadiazine; Trimethoprim | |
| 70 | 236.38 | 1027.39 | Sulfadiazine; Trimethoprim | |
| 71 | 147.56 | 786.75 | Sulfadiazine | |
| 72 | 154.26 | 900.43 | | |
| 73 | 68.62 | 653.82 | | |
| 74 | 119.22 | 955.27 | | |
| 75 | 109.03 | 763.53 | | |

Nd – not determined
